# Exploring the Switchgrass Transcriptome Using Second-Generation Sequencing Technology

**DOI:** 10.1371/journal.pone.0034225

**Published:** 2012-03-29

**Authors:** Yixing Wang, Xin Zeng, Niranjani J. Iyer, Douglas W. Bryant, Todd C. Mockler, Ramamurthy Mahalingam

**Affiliations:** 1 Department of Biochemistry and Molecular Biology, Oklahoma State University, Stillwater, Oklahoma, United States of America; 2 Donald Danforth Plant Science Center, St. Louis, Missouri, United States of America; University of Toronto, Canada

## Abstract

**Background:**

Switchgrass (*Panicum virgatum* L.) is a C4 perennial grass and widely popular as an important bioenergy crop. To accelerate the pace of developing high yielding switchgrass cultivars adapted to diverse environmental niches, the generation of genomic resources for this plant is necessary. The large genome size and polyploid nature of switchgrass makes whole genome sequencing a daunting task even with current technologies. Exploring the transcriptional landscape using next generation sequencing technologies provides a viable alternative to whole genome sequencing in switchgrass.

**Principal Findings:**

Switchgrass cDNA libraries from germinating seedlings, emerging tillers, flowers, and dormant seeds were sequenced using Roche 454 GS-FLX Titanium technology, generating 980,000 reads with an average read length of 367 bp. *De novo* assembly generated 243,600 contigs with an average length of 535 bp. Using the foxtail millet genome as a reference greatly improved the assembly and annotation of switchgrass ESTs. Comparative analysis of the 454-derived switchgrass EST reads with other sequenced monocots including Brachypodium, sorghum, rice and maize indicated a 70–80% overlap. RPKM analysis demonstrated unique transcriptional signatures of the four tissues analyzed in this study. More than 24,000 ESTs were identified in the dormant seed library. *In silico* analysis indicated that there are more than 2000 EST-SSRs in this collection. Expression of several orphan ESTs was confirmed by RT-PCR.

**Significance:**

We estimate that about 90% of the switchgrass gene space has been covered in this analysis. This study nearly doubles the amount of EST information for switchgrass currently in the public domain. The celerity and economical nature of second-generation sequencing technologies provide an in-depth view of the gene space of complex genomes like switchgrass. Sequence analysis of closely related members of the NAD^+^-malic enzyme type C4 grasses such as the model system *Setaria viridis* can serve as a viable proxy for the switchgrass genome.

## Introduction

Even though genome sequencing technologies have become progressively more efficient over the last few years, complete sequencing of complex plant genomes is still technically challenging and cost prohibitive. Identification of transcribed portions of the genome using expressed sequence tags (ESTs) provides a viable alternative for analyzing non-model systems and organisms with large genome sizes, wherein whole genome sequencing is daunting. ESTs have high functional information and have been proven to be valuable for gene annotation and gene discovery [1], [2], [3]. ESTs have been useful for development of molecular markers [4], [5], [6], [7], [8], comparative genomics [9], [10] and for genetic analysis of adaptive traits [11], [12]. *Ipso facto*, genes are expressed in particular tissues or cell types, developmental stages and vary in their expression levels by several orders of magnitude. Traditional EST projects require substantial investments in terms of library construction and sequencing, especially if the goal is to capture rare transcripts [13].

Next generation sequencing (NGS) technologies such as pyrosequencing, bypass lengthy and relatively low throughput steps involved in Sanger sequencing and provide rapid and economical technologies for transcriptomics [14], [15], [16], [17], [18], [19], [20]. To date, the massively parallel DNA sequencing developed by Roche Life Sciences called 454 pyrosequencing is the most widely used next-generation technology for *de novo* sequencing and analysis of transcriptomes of non-model systems. The first commercial NGS platform, the 454 GS20, produced 200,000 reads with an average read length of 100 bases per run [14], [18]. Rapid improvements in emulsion PCR and sequencing chemistry have greatly improved the throughput, read-length and accuracy of 454 sequencing technology [21]. The newest 454-sequencing platform, GS FLX Titanium, can generate a million reads with an average read length of 400 bases at 99.5% accuracy per run.

Switchgrass (*Panicum virgatum*) is a C4 perennial grass selected in 1991 by the Department of Energy as a model herbaceous energy crop for the development of a renewable feedstock resource to produce transportation fuel [22]. This choice has been attributed to several features of this plant native to North America: 1. Biomass – switchgrass plants can grow 3–8 feet tall depending on ecotype; 2. Low input – switchgrass can thrive on marginal lands with minimal input of nutrients and water; and 3. Carbon sink – the large and fibrous root system of switchgrass serves as a major reservoir of captured carbon [22], [23], [24]. To further accelerate the pace of switchgrass breeding several groups have embarked on developing genomic resources including SSR markers [25], [26], [27], [28], ESTs [9], [29] and miRNAs [30].

In this study we conducted 454 based transcriptome analysis in four different switchgrass tissues that are under-represented in the current EST collections – dormant seeds, germinating seedlings, emerging tillers and flowers. We describe the *de novo* assembly of these ESTs, and assembly and annotation of ESTs using the foxtail millet draft genome as a reference. Second, we discuss the transcriptome coverage using proxy methods in the absence of the switchgrass genome sequence. Thirdly, we assessed the expression profiles from these four tissue samples. Fourthly, we examined these ESTs for predicting more than 2000 SSRs that can be very useful for mapping agronomic traits and population genetic studies in switchgrass.

## Results

### 454 sequencing

Four normalized cDNA pools using RNA extracted from dormant seeds, seedlings, tillers and flowers of switchgrass were created. Pyrosequencing of these cDNA pools on a 454 Life Sciences FLX Titanium platform produced approximately 360 million base pairs (Mbp) of sequence data, in the form of 979,903 reads. The cDNA library from dormant seeds had the lowest number of reads (Table 1). The longest read (695 bp) and largest number of total bases sequenced were from the flower sample.

**Table 1:** Raw metrics of 454 sequencing of switchgrass transcriptome.

	Total	Seeds	Seedling	Tiller	Flower
FltrPassWells	979,903	193,511	270,778	246,073	269,541
Total Bases	359,009,624	74,023,325	85,638,441	97,098,266	102,249,592
Avg. length	367	383	317	395	380
Median Len	395	415	331	432	421
Longest Read	695	643	624	652	695

### Filtering and de novo assembly

**Figure 1:**
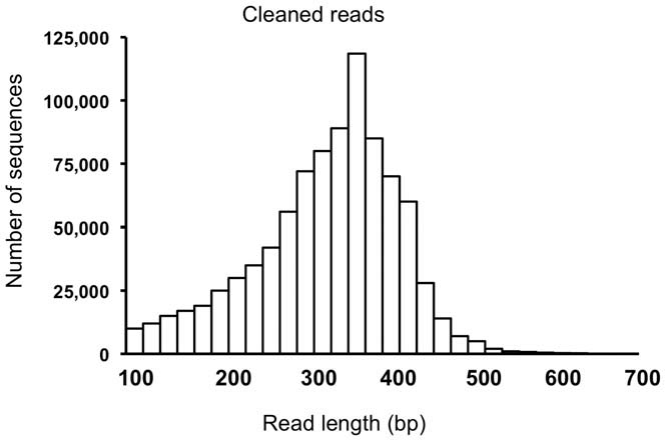
Frequency distribution of 454 sequencing read lengths. Histogram of Roche 454 GS-FLX Titanium read lengths after filtering and trimming adapters.

**Figure 2:**
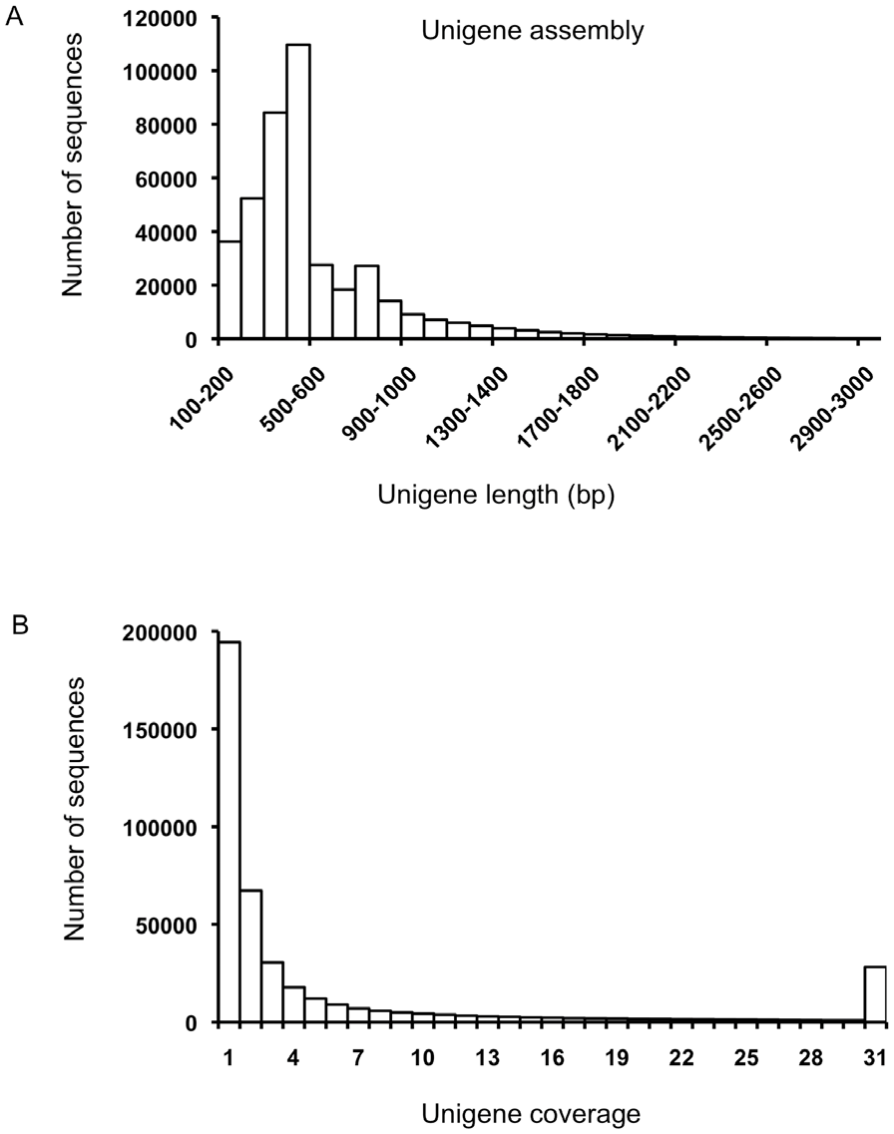
Unigene assembly features of switchgrass transcriptome. (A) Histogram of contig lengths following the 2-step *de novo* assembly process. The x-axis has been truncated at 3 kb. The longest contig is 12,437 base pairs. (B) Histogram of the average read-depth coverage for assembled contigs. Coverage values greater than 30× have been binned together.

Filtering was done to remove poor quality sequence reads, ESTs that were less than 100 bp after trimming the adapters and polyA/T, and ESTs that matched the NCBI prokaryote sequences. Following these filtering parameters, 69,506 reads were removed. The average read length of cleaned reads was 367 bp (Fig. 1). Pre-clustering using a custom BLAST-like alignment tool [31]-based pipeline was conducted with 910,397 454 EST sequences and 545,894 switchgrass ESTs obtained from Genbank, totaling 1,456,291 sequences. Pre-clustering created relatively smaller (than the entire dataset) groups of overlapping/partially-overlapping reads that were then *de novo* assembled. To accommodate the potential for multiple homologs given the polyploidy in switchgrass [32], the clustering and assembly approach allowed for individual ESTs to exist in more than one cluster. The ESTs were assembled into 243,601 contigs, while 215,923 ESTs remained unassembled. The assembled contigs had a mean length of 535 bp (Figure 2A). About 65% of the reads contributed to contigs that were between 200–600 bp long and nearly 87% of the assembled contigs had between 2–50 EST reads (Figure 2B). This highly left-skewed distribution of assembled contigs is typical for normalized libraries and confirmed that cDNA normalization was effective [33].

BLAST analysis was undertaken to estimate the proportions of grass genes represented in the switchgrass transcriptome data. This analysis showed that the switchgrass transcriptome data represents up to 24,675 Brachypodium transcripts (79.5% of the 31,029 Brachypodium v1.2 transcripts analyzed), 28,375 sorghum transcripts (78% of the 36,338 sorghum transcripts analyzed), 41,625 rice transcripts (73% of the 56,797 rice transcripts analyzed) and 69,794 maize transcripts (71.5% of the 97,522 maize genes analyzed). These results are consistent with the broad sampling of the switchgrass transcriptome expected using the 454 pyrosequencing technology, assuming that although switchgrass is polyploid the gene space is not very different from other grasses.

### Reference based assembly

**Figure 3:**
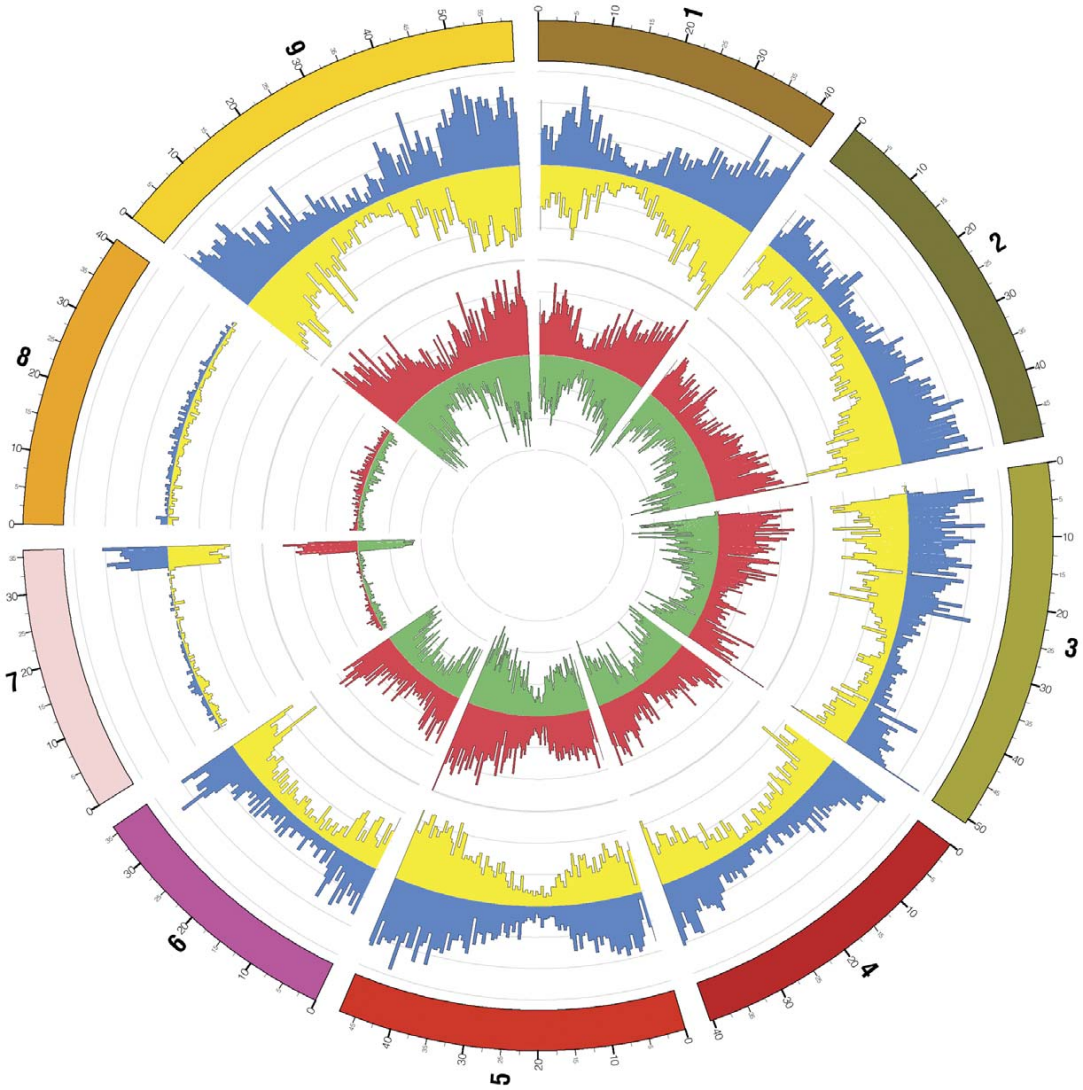
Switchgrass 454-based sequencing reads mapped to the foxtail millet genome. The number of contigs and unassembled ESTs that produced significant alignments to the foxtail millet genome are plotted for each 0.5 megabase interval. Radial axis line represents one log interval. Numbers on the circumference represent the Foxtail millet chromosomes (1–9) and each chromosome is given a different color. Assembled contigs aligned to the forward strand of the *Setaria italica* reference genome assembly are shown in blue for forward strand alignments and in yellow for reverse strand alignments. Singleton reads aligned to the forward strand are shown on the same figure in red, while singleton reads aligned to the reverse strand are shown in green. Diagram was prepared using Circos (http://mkweb.bcgsc.ca/circos).

Availability of the foxtail millet genome sequence (http://www.phytozome.net) prompted a reference guided-sequence assembly of the switchgrass ESTs. Foxtail millet is the closest member of the Panicoideae subfamily with a sequenced genome and a shared common ancestry with switchgrass between 7–10 million years ago [34], [35], [36]. This analysis yielded 98,086 assembled contigs and 82,902 unassembled ESTs. In the Setaria genome assembly the nine scaffolds corresponding to the nine chromosome pseudomolecules comprise nearly 99% of the total sequence length (http://www.phytozome.net). The assembled contigs and the unassembled ESTs were assigned to the foxtail millet genome in 0.5-Mb intervals based on BLAST similarity scores. Biases in the distribution of the ESTs in the central portions of each chromosome in the pericentric regions were obvious (Figure 3). Curiously, the representation of the assembled contigs as well as unassembled ESTs on the foxtail millet chromosomes 7 and 8 was extremely low.

### Gene Ontology (GO) Annotations

**Figure 4:**
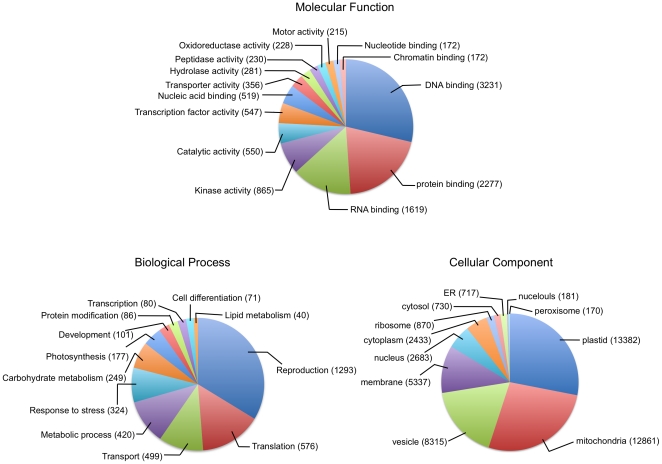
Plant GO-slim terms associated with switchgrass. Venn diagram of the distribution of plant GO-slim terms associated with switchgrass contigs represented in molecular function, biological process and cellular component categories.

Plant specific GO slim terms associated with 36,080 (36.7%) of the 98,086 assembled EST contigs were available. Of these, assignment of contigs to the cellular component made up the majority (49,028), followed by biological process category (12,075) and molecular function category (3916). The GO categories represented in the switchgrass transcriptome did not show any significant biases and showed similar distribution patterns reported in other plant species [12], [37], [38]. The majority of contigs with annotations for cellular component category were in plastids or mitochondria, but a large number were also associated with vesicles and membrane (Figure 4). Predominant contig annotations for the biological processes category were reproduction, followed by translation, transport and response to stress (Figure 4). DNA binding, RNA binding and protein binding were the major GOs associated with the molecular function (Figure 4).

### Transcriptome coverage

Since the sequence of the switchgrass genome is unavailable, the actual size and composition of the transcriptome is not known. We used a simulation-based tool, ESTcalc [13], to approximate the coverage of switchgrass transcriptome using 454-pyrosequencing data. Based on this simulation analysis, our dataset covers 92% of the transcriptome, with every gene represented by at least one read (Table 2). We compared the NCBI switchgrass Unigene set containing 20,973 genes with the ESTs from our 454 analyses. BLAST analysis indicated that 20,963 of the 20,973 (99.95%) Genbank unigenes are represented by 125,341 contigs derived from the current 454 assemblies. It should be noted that our assembly process allows individual ESTs to belong to more than one EST cluster. This approach is intended to reduce erroneous assembly of homologs, paralogs, and splice variants, but results in more contigs than the approach used by dbEST for generating the unigenes.

**Table 2:** ESTcalc-based transcriptome coverage estimates.

Input parameters	ESTcalc	Actual
Number of technologies	1	1
Technology	454 GS-FLX	454 GS-FLX (Titanium)
Library type	normalized	normalized
Reads/plate	979,903	979,903
Mean read length (bp)	367	366.56
**Predicted assembly**		
Total assembled sequence (MB)	359.6	279.9
Unigene count	28665	523228
Mean contig length (bp)	963	535
Mean contig length (longest contig per gene, bp)	1274	-
Singleton yield (%)	13	15
Percent transcriptome (%)	92	-
Percent of genes tagged (%)	100	-
Percent of genes with 90% coverage (%)	80.3	-
Percent of genes with 90% coverage by largest contig (%)	68	-
Percent of genes with 100% coverage (%)	31.7	-
Percent of genes with 100% coverage by largest contig (%)	30.1	-

Ultraconserved orthologs (UCOs) and APVO (*Arabidopsis thaliana*, *Populus trichocarpa*, *Vitis vinifera* and *Oryza sativa*) sequences represent a highly conserved set of genes expected to be present in eukaryotic and plant genomes, respectively, and has been used as a proxy for gene detection and sampling breadth [38]. We identified all the 357 (100%) UCOs in assembled switchgrass contigs. We detected 878 (91.5%) of the 959 shared single copy tribes represented in the PlantTribes database [39], [40]. [38] Based on these estimations and comparisons we estimate that the set of switchgrass ESTs identified in this study has covered more than 90% of the switchgrass gene space.

### Assessment of repetitive sequences in the switchgrass transcriptome

#### i. Retrotransposon abundance

Given that switchgrass has a polyploid genome we examined the abundance of retrotransposon sequences in the EST collection. It has been reported that retrotransposons constituted nearly 7.5% of the sequenced genome of sorghum [41] and a large number of copia-like and gypsy-like retrotransposons were actively transcribed in sorghum protoplasts derived from embryogenic callus tissues [42]. We retrieved the sorghum sequences for the 24 copia-like and 48 gypsy-like elements to develop a query database. We identified 8826 assembled switchgrass EST contigs (4.06%) and 6990 unassembled EST sequences (4.24%) that showed significant homologies to the 74 sorghum retrotransposon sequences. Retrotransposon abundance from large EST collections from 10 different plant taxa ranged between 0.03–0.1% [12]. In a recent study in the *Pinus contarta* transcriptome using 454 pyrosequencing, retrotransposons constituted 3.89% of the ESTs [12]. This clearly suggested that as in *P. contorta*, in switchgrass there is a significant over-representation of retrotransposons and the fact that these were identified from RNA samples indicates that they are actively transcribed in the tissues analyzed in our experiments.

#### ii. Simple Sequence Repeats (SSRs)

**Figure 5:**
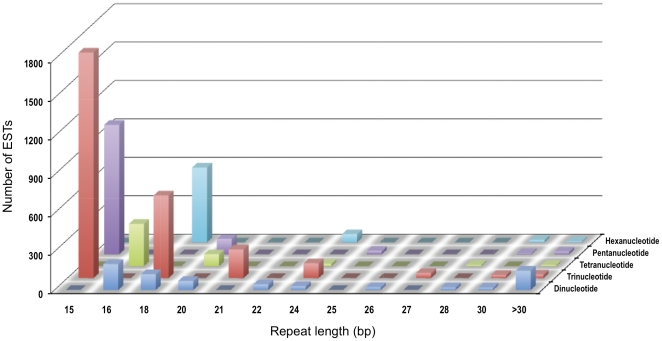
Distribution of simple sequence repeats in switchgrass ESTs. Di-, tri-, tetra-, penta- and hexa-nucleotide repeats were analyzed and their frequency plotted as a function of the repeat number.

The assembled switchgrass contigs with annotations were used for identifying SSRs. The distribution of di-, tri-, tetra-, penta- and hexa-nucleotide SSRs in these assembled contigs are shown (Figure 5). This analysis using the 243,600 assembled switchgrass contigs with annotations, identified 21,437 contigs that contained SSRs between 2–6 nucleotides and greater than 15 bp in length using PHOBOS program. This indicated that nearly 8.8% of the switchgrass ESTs contained SSRs and this is 2.8 times more than the average number of EST-SSRs reported in other grasses [43]. The results from the PHOBOS output were filtered in excel to identify only the perfectly matching SSRs. A total of 5840 perfect di-, tri-, tetra-, penta-, and hexa-nucleotide SSRs longer than 8, 6, 4, 3, and 3 repeat units, respectively, were identified (Figure 5). The tri-nucleotide repeats were the most abundant (48.6%), which is consistent with the findings in other grasses including switchgrass 9,43. The identified SSRs were GC rich (Table 3). The CCG tri-nucleotide repeats were the most abundant (22%) class of repeats that was identified in this study (Figure 5) and has been reported in earlier reports on other grass species [43]. Among di-nucleotide SSRs, AG repeats were the most abundant while the CG repeats were the least abundant. Interestingly, we identified that the frequency of penta-nucleotide repeats (20%) were more abundant than di-nucleotide repeats (11%) among these EST sequences.

**Table 3:** Commonly found SSR repeat units in switchgrass ESTs.

Repeat unit	Reads	Repeat unit	Reads
AG	337	CCCCG	28
AT	134	ACACG	27
CG	11	ACCGG	26
CCG	1279	AATGC	25
AGC	417	ACCGG	26
AGG	282	AATGC	25
AAG	244	AAGAG	22
ACC	165	AGCTC	19
AAC	142	AGGCG	19
ACG	140	CCCGG	19
ATC	94	AAGGG	18
AAT	44	ACCCG	18
ACT	30	ACAGC	16
ACAT	45	AGATC	16
AAAT	34	ACACC	15
AGGG	34	AATGG	14
CCGG	30	AGCGG	14
CCCG	28	ATCCG	14
AAAG	25	AAAGG	13
AGCT	25	AAATC	13
AGCG	21	ACGGC	13
ACGC	20	AGATG	13
AAAC	19	AAAGC	12
AGGC	19	AACCG	12
ATCC	17	ACTCC	12
AAGC	16	AATCC	11
AGCC	15	ACGCC	10
AGAT	14	ATCCC	10
AATC	11	ATCGC	10
ACCC	11	CCGGCG	47
AAGG	10	ACGGCG	24
ATGC	10	AAGCCG	23
AAAAG	106	AGGCGG	20
CCGCG	65	AGCTCC	17
AAACC	44	AAGAGG	16
AAAAC	43	ACCGCC	16
AAAAT	41	AGAGGG	15
AAATT	35	CCCCCG	15
AGGGG	32	AAGGAG	12
AGCCG	31	AGCGGG	12
AGAGC	30	AGCGGC	11
AGAGG	29	ACGAGG	10

### Expression profiling

**Figure 6:**
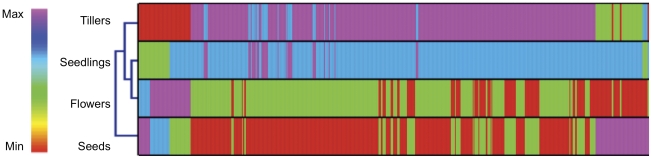
Heat map of switchgrass gene expression in four different tissues. Individual columns represent the four tissues used in this study while each row represents a unique contig. The dendrogram represents the similarity of expression profiles between the tissue samples based on average linkage clustering. The color key represents the log2 transformed RPKM values. Red indicates a low level of expression, green and blue are intermediate levels, while purple indicates maximal levels of gene expression. The highest value for RPKM after log 2 transformation was 17 and the lowest value was 0.1.

Reads per kilobase per million mapped reads (RPKM) values were obtained for 240,981 assembled contigs. In this analysis 1 RPKM corresponds to ∼25 mapped reads per kilobase of target transcript sequence. A total of 44,279 contigs had RPKM values of less than 1 and hence were not considered for further analysis. Of the 196,702 contigs, 120,193 (61%) were from flower library, 102,184 (51.9%) from germinating seedlings library, 86,128 (43.8%) from tiller library and 80,923 (41.1%) from dormant seed library. We chose to examine contigs whose RPKMs were greater than ten in at least one of the four tissues analyzed. This resulted in 136,612 contigs used for clustering analysis. The RPKM values were log 2 transformed and subjected to average linkage hierarchical clustering using the Genesis software [44]. This analysis showed that the transcriptional landscapes of the four tissues that were examined were unique (Figure 6). A majority of transcripts showed maximal expression in the tiller tissue, while in the seedling tissues the expression of these genes was at an intermediate level. In contrast to the tiller tissues, expression levels of a majority of dormant seed ESTs were low. Surprisingly, we identified several clusters of genes that had their highest expression in dormant seeds. In the flowers, a majority of the ESTs showed low to intermediate levels of gene expression when compared with tillers and seedlings.

### RT-PCR analysis

**Figure 7:**
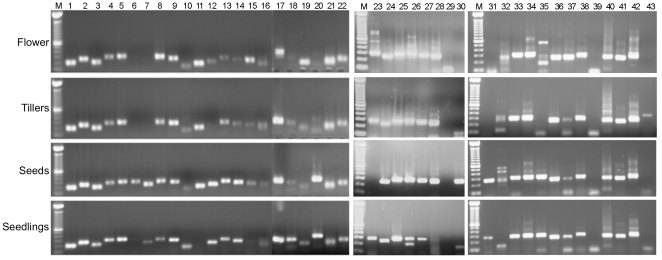
Switchgrass gene expression analysis by RT-PCR. The four panels represents the amplifications from the cDNA derived from the four different tissues – flowers, tillers, seeds and seedlings. The lanes labeled 1–22 are amplifications of EST contigs with functional annotations. Lanes 23–42 contains amplifications of EST contigs that did not show any homologies to sequences in the databases. Lane 43 is the amplification of the teosinte branched 1 gene that shows tiller specific expression. M indicates the 100 bp DNA size ladder.

We examined the gene expression patterns of 22 genes with annotations derived from homology searches and also a set of 20 genes that did not show any homologies to any of the sequences in the various databases (Figure 7). Primers were designed from the EST sequences to amplify 200–300 bp products for most of the selected genes (except lanes 29 and 39 wherein the EST sequence was about 125 bp). Most of the amplifications resulted in single discrete product and two of them resulted in multiple bands in only certain tissues. Even though this was not a quantitative PCR analysis we observed that the gene expression patterns were significantly different for several genes among the four tissues tested here. All of the amplifications from the EST sequences with no homologies gave the expected size amplification product using the switchgrass cDNAs from the four different tissues. This analysis confirmed that these novel EST sequences were indeed expressed in switchgrass tissues.

### Gene inventories

We focused our analysis of the switchgrass transcriptome on genes associated with C4 photosynthesis, an attribute that is extremely important for biomass accumulation.

#### C4 photosynthesis

**Figure 8:**
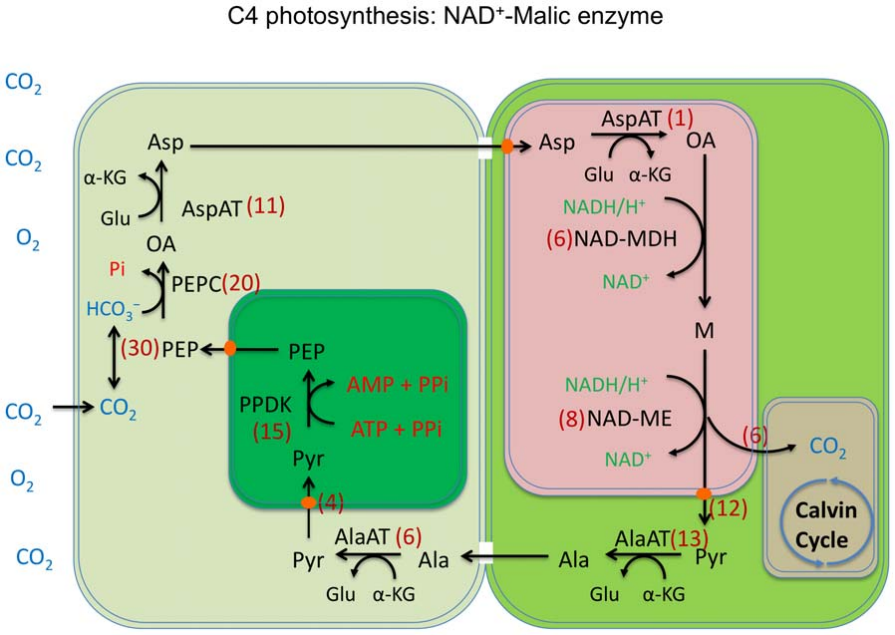
Overview of the C4 NAD^+^-Malic enzyme photosynthesis. The numbers shown in red font in the figure correspond to the ESTs that were identified in the 454 sequencing analysis. Ala-Alanine; AlaAT – Alanine aminotransferase; Alpha-KG-alpha ketoglutarate; Asp-aspartate; AspAT-aspartate aminotransferase; CA-carbonic anhydrase; Glu-glutamate; OA-oxaloacetate; PEP-phosphoenol pyruvate; PEPC-phosphoenolpyruvate carboxylase; PPDK –pyruvate phosphate dikinase; Pyr-pyruvate; NAD^+^-MDH- NAD^+^ dependent malate dehydrogenase; NAD^+^-ME- NAD^+^ dependent malic enzyme.

Based on GO annotations derived from homology searches we have identified all major enzymes associated with C4 photosynthesis (Figure 8). Among the C4 pathway genes in our collection, ESTs coding for carbonic anhydrases formed the largest group. Multiple sequence alignments juxtaposed with the GO annotations suggested that there are probably five different genes encoding carbonic anhydrases in switchgrass. This estimate of the number of carbonic anhydrase genes is consistent with an earlier report in switchgrass [9].

Phosphoenolpyruvate carboxylase (PEPC) was the second most abundant enzyme of the C4 pathway based on EST representation. Based on multiple sequence alignment analysis we speculate that there are three PEPC gene families in switchgrass with reference to their putative localization – cytoplasm, mitochondria or plastids. Recently, rice PEPC gene localized to the chloroplasts has been shown to be important for ammonium assimilation [45]. It will be important to examine the PEPC gene expression in switchgrass in the context of its higher nitrogen use efficiency compared with other grasses.

Pyruvate is an important metabolite especially with reference to the CO_2_ concentrating mechanisms in C4 plants like switchgrass [46], [47]. Recently it was shown that sodium-dependent pyruvate transporters in plastids were encoded by BILE ACID: SODIUM SYMPORTER FAMILY PROTEIN (BASS) [48]. Sixteen ESTs in our collection were identified showing various levels of homologies to BASS protein. Four of them are possibly localized to plastids based on their GO annotations and showed closest homologies to BASS2 and BASS4 of C4 *Flaveria* species identified in the above study. Interestingly, four ESTs were annotated as being localized to mitochondria and eight other ESTs were predicted to be associated with both plastids and mitochondria. Multiple sequence alignment analysis indicated that ESTs with dual localization were unique and did not cluster together with plastidial or mitochondrial pyruvate transporters (Figure S1). Further detailed analysis of expression patterns and precise *in situ* localization of putative mitochondrial and plastidial pyruvate transporters warrants attention.

In all the C4 plants with NAD^+^- malic enzyme (NAD^+^-ME) this enzyme is localized to the mitochondria. Consistent with these observations we found that all eight ESTs with strong homologies to the NAD^+^-ME were annotated as being localized to the mitochondria. Curiously, we identified four ESTs that were annotated as having malic enzyme activity but were localized to cytoplasmic membrane bound vesicles. Multiple sequence alignments indicated that these four ESTs were unrelated to the canonical NAD^+^-ME ESTs (Figure S2).

Bundle sheath mitochondria and mesophyll cell cytosol are the major locations for the Aspartate aminotransferases in C4 plants [49]. A majority of Aspartate aminotransferases identified in this study was predicted to be cytosolic while only one was identified as being localized to mitochondria. Alanine aminotransferases that lead to reversible conversion of pyruvate to alanine were abundantly expressed in the tissues analyzed in this study. In our collection, 20 ESTs predicted to represent genes with alanine transaminase activity were identified. Based on their consensus sequences we estimate that this gene family may be represented by 5–6 unique genes. More than 20 malate dehydrogenase ESTs were identified in our collection. Only six of these were localized to mitochondrion while six were localized to both mitochondria and chloroplasts, and 10 were localized to the cytoplasm based on their GO annotations. Even the six localized to the mitochondrion were quite different and could represent four unique genes based on the multiple sequence alignment analysis.

#### Seed dormancy associated genes

Most of the large-scale transcriptome and proteome studies in seed dormancy have been conducted in the model plant *Arabidopsis thaliana*. Previous studies using DNA microarrays indicated that there are about 12,000 stored mRNAs that were detectable in dry seeds of Arabidopsis [50] and barley [51], and 17,000 genes in rice [52]. Using the 454 platform we have identified 24,095 contigs expressed in the dormant seeds of switchgrass [50], [51], [52]. Genes from all major GO categories were identified in the dormant switchgrass seeds.

We carefully analyzed one study wherein the highly dormant Arabidopsis ecotype Cvi was used for microarray analysis [53]. In a comparison of the dormant Cvi seeds versus ripened seeds, 442 genes were identified as being differentially expressed and up regulated in the former. We compared this list of 442 genes with the genes showing highest expression in the dormant switchgrass seed sample. We identified 170 genes in the switchgrass dormant seed samples that were closely related to the 442 Arabidopsis genes associated with dormancy (Table S1). The more than 38% overlap in the switchgrass and Arabidopsis genes suggest that the genetic mechanisms leading to dormancy are comparable between monocots and dicots. Nearly one fourth of the genes in the Arabidopsis data set are still annotated as unknown proteins or expressed proteins with unknown functions. We speculate that as these annotations are updated, the extent of overlap will improve significantly. It will be interesting to examine which of these stored mRNAs are actually translated and are crucial for maintaining dormancy. Using captured polysome-associated RNAs in combination with high-throughput transcript sequencing will be a useful strategy to explore this issue further.

Of the 53 different transcription factors (TFs) in the Arabidopsis dormancy related gene set [53], homologs to 30 were identified in the switchgrass 454 EST data. This included members of a DREB subfamily, AP2 domain containing TFs, several different classes of zinc finger TFs such as C2H2, C3H, C5HC2, NF-YA, ethylene responsive factors such as EIN3, ERF, auxin responsive TFs such as ARF, Myb family TFs, Scarecrow proteins, NAC, NAM, Aintegumenta, bZIPs, and MADS box TFs.

Ten different heat shock proteins including two heat shock transcription factors were also identified in this set. Another set of interesting genes was the RNA binding proteins implicated in post-transcriptional gene regulation and included Mei2, pumilio, RRM domain containing proteins, and the decapping enzyme. Genes encoding various protective proteins such as late embryogenesis abundant proteins, GSTs, Mn SOD, glutathione gamma-glutamylcysteinyltransferase important for GSH biosynthesis, peroxiredoxin, thioredoxin, and PMSR were also identified in the dormant seeds.

## Discussion

Expressed sequence tag analysis is one of the most popular techniques for gene discovery. Traditional EST analysis by Sanger sequencing is still very time-consuming, labor and cost intensive. The advent of the next generation sequencing (NGS) technologies has circumvented many of the pitfalls associated with the conventional EST analysis. Apart from the speed and the cost, NGS eliminates the bacterial cloning step that can bias the composition of the cDNA libraries. The Roche GS FLX NGS platform has proven to be valuable for non-model plant systems such as olive [54], chestnut [55], *Artemisia annua* [20], ginseng [8], strawberry [56], bracken fern [38] and recently, in switchgrass [29].

In this study four different tissues were analyzed based on their agronomic importance and/or their under-representation in existing EST collections. Dormancy is one of the major agronomic problems with reference to large-scale production of switchgrass directly from seeds [57]. There are no studies to date that examine the switchgrass genes associated with dormancy. Flower tissue is under-represented in the public switchgrass EST databases. Tillering is an important trait that has a direct bearing on the biomass yield in switchgrass [58]. Furthermore, young tiller tissues are under-represented in existing EST collections.

### Assembly quality

The 3.8×10^8^ bp of sequence data here represents a substantial sequence resource and nearly doubles the expressed sequence data available for switchgrass in Genbank (NCBI Genbank dbEST). The increased read lengths (average of 367 bp) from the 454 GS Titanium instrument helped to assemble contigs that were approximately 535 bp that is much larger than the studies that used previous version of the 454 technologies [6], [10], [59]. *De novo* assembly of the switchgrass ESTs indicated that about 85% of the ESTs could be assembled into contigs while 15% remained as singletons. The higher success rate with the *de novo* assembly may be due to the iterative assembly process used in this study. Using the foxtail millet genome as a reference reduced the unassembled ESTs to approximately 8%. This clearly demonstrates the value of using a closely related species as a surrogate reference in the absence of a whole genome sequence for switchgrass.

### Transcriptome coverage

Estimating the number of genes and the extent of gene coverage is an important metric for transcriptome sequencing projects. In the absence of a genome assembly it is only possible to make an approximation of the extent of coverage. Reciprocal BLAST analysis between switchgrass ESTs from this study and four other monocots (Brachpodium, rice, sorghum and maize genomes) indicate that homologs for 70–80% of the genes in those species are represented in this collection. Even though the sorghum genome is about 75% larger than the rice genome, it has been reported that these two grasses have similar quantities of euchromatin (252 Mb and 309 Mb, respectively) [41]. On the same grounds, we speculate that a significant proportion of the switchgrass gene space has been covered by this current study and based on the ESTcalc estimations this may be as high as 90%.

In a previous EST analysis in switchgrass using the conventional Sanger sequencing technology, sorghum was used as the reference genome. Switchgrass ESTs were evenly distributed across the sorghum pseudomolecules [9]. In this current study using the foxtail millet genome as reference we observe that representation of switchgrass ESTs on the pseudomolecules 7 and 8 of the *Setaria italica* genome is conspicuously low. We speculate this may be due to vast stretches of repetitive sequences on these chromosomes that may have been masked during the assembly process and in turn led to this skewed distribution. It is also possible that chromosomes 7 and 8 of the *S. italica* genome may have diverged significantly. A complete analysis of the *S. italica* genome sequence will provide more insight in this regard.

### Marker development

EST-based SSR markers are advantageous when compared with genomic SSRs owing to their higher PCR amplification rates and cross-species transferability [60]. In a previous EST analysis study in switchgrass 830 SSRs were successfully amplified and about 38% of these were reported to be polymorphic between the parents of a mapping population [9]. We conducted a BLAST analysis with our EST data sets and the primer sequences reported from the above study. We found that less than 5% of the primer sequences showed perfect matches to the sequences in our collection (Wu and Mahalingam, unpublished data). It is possible that the use of different cultivars in the two studies may be one of the reasons for this low degree of overlap. Our ongoing work is assessing amplification efficiency and polymorphism rates for more than 1000 EST-SSRs using the parents of a mapping population. The frequency of SSRs identified in Next-Gen sequencing projects depends on template used, criteria for defining SSRs, and the software used for identifying SSRs in the sequences, and has shown extensive variation in recently reported studies [12], [61], [62]. Despite these variations, the above-mentioned work and the current study illustrate the speed and cost effectiveness of identifying SSRs in non-model systems using the 454 technology.

In summary a highly significant improvement in the switchgrass EST assembly was facilitated by the availability of the foxtail millet draft genome. The 180,000 unique sequences (98,000 assembled contigs and 82,000 unassembled ESTs) identified in this 454-based EST collection represent a major genomic resource for switchgrass. We estimate that more than 90% of the gene space of switchgrass is represented in this analysis. Identification of more than 24,000 unique sequences in the dormant seeds of switchgrass was unexpected and provides an important resource to further investigate this important agronomic trait. The large number of EST-SSR markers identified in this study will provide valuable resources for marker-assisted breeding programs. Sequencing the transcriptomes and genomes of closely related members of the NAD^+^-malic enzyme- type C4 grasses such as the model system *Setaria viridis* is extremely important and will be a viable proxy for the switchgrass genome [63].

## Materials and Methods

### Sample collection

‘Cimarron’, a high biomass yielding switchgrass cultivar released by Oklahoma State University was used for this analysis. Seeds were first placed on a Whatman filter paper pre-soaked in 50% Benomyl solution, a fungicide. Seeds were surface sterilized in Falcon tubes containing 30 ml isopropanol. Then seeds were placed in 50% bleach solution for 5–10 mins. Seeds were washed in distilled water for 4–5 times to remove the bleach. Sterilized seeds were placed on top of a Whatman paper pre-soaked with 50% Benomyl in a petri dish. Petri dishes were placed in a growth chamber with 10 hours/day and 14 hours/night for six days. Seeds that failed to germinate were harvested separately and labeled as dormant seeds while those that had sprouted were harvested as germinating seedlings. Samples were frozen in liquid nitrogen and stored at −80°C. Seeds of SL93-7×15 were planted in cones containing SUNGRO metromix 200 series soil in the Plant and Soil Sciences greenhouse facility at Oklahoma State University. Plants were maintained at 24°C with 16 h day and 8 h night regime. Six weeks following germination, the young emerging tillers were harvested, frozen in liquid nitrogen and stored in −80°C. Flowers were harvested from field-grown SL93- 7×15 plants in the Oklahoma State University Agricultural station agronomy plots, Stillwater, OK.

### RNA isolations

About 100 mg of frozen switchgrass seed tissue was ground to fine powder with liquid nitrogen and sterile quartz powder. The powder was transferred to a 2-ml tube with 2 ml of extraction buffer (8 M LiCl, 2% b-mercaptoethanol, pre-cooled to −20°C) and was incubated overnight at 4°C. The mixture was centrifuged at 13,000 rpm for 30 min at 4°C. The resulting supernatant was decanted, and the pellet was washed with cold (4°C) 70% ethanol, briefly air-dried, and dissolved in 1 ml of solubilization buffer (0.5% SDS, 100 mM NaCl, 25 mM EDTA, 10 mM Tris-HCl, pH 7.6, 2% 2-mercaptoethanol). Following this, RNA was further purified, once with phenol: chloroform: isoamyl alcohol (25∶24∶1), and twice with chloroform: isoamyl alcohol (24∶1). The aqueous phase was precipitated with 0.1 volumes of 3 M sodium acetate and 1.5 volumes of ethanol.

The tubes were then centrifuged at 13,000 rpm for 30 min at 4°C, the supernatant was poured off, and 0.5 ml of 3 M sodium acetate was added. The pellet was vortexed for 1 min and centrifuged at 13,000 rpm for 10 min at 4°C, washed with 70% ethanol, and dissolved in 50 ul of RNase free water. Total RNA from switchgrass flowers and tillers was extracted using the RNEasy Plant Mini kit (Qiagen). The RiboMinus™ Plant kit for RNA-Seq (Invitrogen) was used to remove rRNA from all the four samples. All steps were performed according to the manufacturer's instructions. The RNA integrity was assessed by agarose gel electrophoresis.

### cDNA library construction and 454 sequencing

Approximately 200 ng of Ribominus RNA was used for first and second strand cDNA synthesis as described in the cDNA Rapid Library Preparation Method manual (Roche Life Sciences, Inc.) with slight modification. The double stranded (ds) cDNA was nebulized using 30 psi nitrogen for 30 seconds to generate library fragments of the correct size followed by purification in a Qiagen MinElute column. The ds cDNA was eluted in 16 ul Tris-HCl, pH 7.5. The nebulized ds cDNA was used for fragment end repair followed by adaptor ligation. AMPure beads were used for removing the small fragments. Library quantitation was done using TBS fluorimeter and Roche Rapid Libary (RL) standards that are control ds DNA fragments with an attached FAM moiety. The units of the RL standards were “molecules/ul”. The A adaptor on all ds cDNA fragments contained a FAM moiety as well. The RFU value of the library (x) was applied to the RFU values of the standard curve, Y = mx+b, solved for y to yield molecules/ul. Small volume emulsion (em) PCR was set up for each ds cDNA library based on each library's optimal “library molecules/DNA capture bead” that was calculated as described in the emPCR Method Manual- Lib-L SV (Roche Life Sciences, Inc.). After the emPCR, all reactions were pooled followed by capture of the “enriched beads”. Lastly, 790,000 of the enriched beads from each library were loaded into a four-region 454 Life Sciences Picotiter plate and sequenced with “454 Life Sciences FLX Titanium Chemistry”.

### 454 EST filtering and assembly

Roche/454 EST sequences were prepared for assembly by removal of library adapter sequences using estclean (https://sourceforge.net/projects/estclean/) and a custom perl script. Contaminating vector and microbial sequences and poly A/T stretches were removed using SeqClean (http://compbio.dfci.harvard.edu/tgi/software/). Public switchgrass ESTs (546,245) were downloaded from Genbank and filtered for adapters, contaminating vector sequences and poly A/T stretches using estclean and SeqClean. ESTs were pre-clustered using a custom BLAT-based pipeline. Briefly, an all-versus-all comparison was performed using BLAT [31]. A custom perl script was used to generate clusters of overlapping ESTs from the resulting BLAT alignments, allowing for individual ESTs to exist in more than one cluster in order to accommodate polyploidy in switchgrass and avoid mis-assembly of close homologs and paralogs and splice variants. The resulting EST clusters were assembled using iterative cycles of MIRA (http://www.chevreux.org/projects_mira.html) [64] and CAP3 (http://seq.cs.iastate.edu/cap3.html) [65]; four cycles of MIRA assembly were performed and then followed by one cycle of CAP3 assembly performed on the contigs generated by MIRA.

### Descriptive annotations and GO classifications

Descriptive annotations and GO classifications were derived by comparing the assembled switchgrass EST contigs to public sequence databases using NCBI BLAST [66]. Databases used for sequence comparisons were as follows: Brachypodium distachyon v1.2 (downloaded from MIPS; ftp://ftpmips.helmholtz-muenchen.de/plants/brachypodium/v1.2/); Sorghum bicolor v1.4 (downloaded from Phytozome; ftp://ftp.jgi-psf.org/pub/JGI_data/phytozome/v6.0/Sbicolor/); Zea mays ZmB73_4a.53 (downloaded from Gramene; http://www.gramene.org/info/data/ftp/index.html); Oryza sativa v6.1 (downloaded from MSU; ftp://ftp.plantbiology.msu.edu/pub/data/Eukaryotic_Projects/o_sativa/annotation_dbs/); Plant Gene Indexes (http://compbio.dfci.harvard.edu/tgi/plant.html); Genbank nr protein database (ftp://ftp.ncbi.nih.gov/blast/db/); and Genbank est_others database (ftp://ftp.ncbi.nih.gov/blast/db/). EST contigs were compared to the nucleotide sequence databases listed above using BLASTN with default settings. Unassembled ESTs were compared to the Plant Gene Indexes (blastn; http://compbio.dfci.harvard.edu/tgi/plant.html) using BLASTN with default settings. Putative orphan transcript contigs that had no matches to plant sequences were further compared to the Genbank nr protein database using BLASTX and Genbank est_others database using BLASTN. In all cases, a perl script was used to filter for the single best database match (according to the BLAST bit score) for each query EST or EST contig sequence, with no additional filtering. Gene ontology (GO) classifications for biological process, molecular function and cellular localization were derived from the Plant Gene Index BLAST matches, providing putative GO annotations for only those switchgrass EST contigs matching Plant Gene Index sequences that have GO annotations.

### Reference guided assembly

Switchgrass EST contigs and singletons were aligned to the *Setaria italica* draft genome (v1.64; ftp://ftp.jgi-psf.org/pub/JGI_data/phytozome/v7.0/Sitalica/) using BLAT and best matches were defined as the alignment for each EST contig or singleton with the highest BLAST score. A custom perl script was used to generate clusters of overlapping ESTs from the resulting alignments. The resulting EST clusters were assembled using iterative cycles of MIRA (http://www.chevreux.org/projects_mira.html) [64] and CAP3 (http://seq.cs.iastate.edu/cap3.html) [65]; four cycles of MIRA assembly were performed and then followed by one cycle of CAP3 assembly performed on the contigs generated by MIRA.

### Circos analysis

Assembled contigs and singleton reads were aligned to the Setaria reference genome assembly using BLAT. Contig alignments were mapped to the outermost set of axes, minimum average coverage −0.1, and maximum average coverage 0.1. EST singleton alignments were mapped to the innermost set of axes, minimum average coverage −0.045, and maximum average coverage 0.045. In all cases the per-base alignment depth was averaged over 500,000 base pairs.

### EST expression level estimates

Relative expression levels of 454 ESTs from the four libraries were quantified by a reads per kilobase transcript per million reads (RPKM) analysis. A Perl script was used to shred the Roche/454 ESTs into simulated 40mer Illumina-like RNA-seq “reads”. The 40-mer reads were mapped to the switchgrass EST contigs using SOAPaligner (http://soap.genomics.org.cn/soapaligner.html) [67], and match counts were converted to RPKM values using a perl script. ESTs with RPKM values greater than 10 in at least one of the four tissues were used for constructing heatmaps using GENESIS [44]. RPKM values were log2 transformed. Average linkage clustering was selected.

### Assessment of transcriptome coverage

Three different assessment tools were used to estimate transcriptome coverage. A web based tool called as ESTcalc [13] was used to estimate the predicted transcriptome coverage. Input parameters for the ESTcalc were one for the 454 GSFLX sequencing technology used, 979,903 for the number of reads and 367 bp for the read length. To determine the number of eukaryotic ultra conserved orthologs (UCOs) in the switchgrass 454 transcriptome dataset we used tblastx to query the list of 357 UCO protein sequences from Arabidopsis (sequences available at http://compgenomics.ucdavis.edu/compositae_reference.php) with an e-value threshold of 1e-10. Blast results were parsed to determine the number of switchgrass ESTs that showed a positive hit to the UCO sequences with amino acid alignments of at least 30 residues. We assessed the transcriptome coverage by comparing the switchgrass ESTs with the PlantTribes database [39], [40]. In this analysis 959 shared single copy tribes from *Arabidopsis thaliana*, *Populus trichocarpa*, *Vitis vinifera* and *Oryza sativa* were compared with the switchgrass EST reads using tblastx and an e-value cutoff of 1e-06.

### Estimating retrotransposon abundance

We used BLASTn to search the 454 EST contigs and singletons for 24 families of *copia*-like elements (GenBank accession numbers: CG026188–CG026196 and CF417056–CF417070) and 48 families of *gypsy*-like elements (GenBank accession numbers: CG425584–CG425599 and CF542191–CF542222) that were reported in the sorghum genome [42]. An *E* value threshold of 1e-06 was used for this analysis. Custom perl scripts were used to make these comparisons and to parse and filter the ESTs that contained the retrotransposon sequences.

### RT-PCR analysis

Primers were designed for 57 switchgrass EST contigs (Table S2).

Total RNA (5 ug) was used to make cDNA using Super-Script III Reverse Transcriptase (Invitrogen). PCR amplification was performed using Taq Master Mix Kit (Qiagen). Following initial denaturation at 95°C for 3 min, the PCR reaction was carried out in a PTC-200 thermal cycler (MJ Research) under the following conditions: denaturation at 94°C for 30 s, annealing at 60°C for 30 s, and extension at 72°C for 1 min. The final extension was carried out at 72°C for 10 min. Reaction products and DNA size markers (100 bp DNA ladder, Invitrogen) were resolved on the 1% agarose gels and visualized under UV light following ethidium bromide staining.

### SSR analysis using PHOBOS

The GUI-based PHOBOS software (Phobos 3.3.11, 2006–2010, http://www.rub.de/spezzoo/cm/cm_phobos.htm) was used for SSR analysis. The minimum repeats unit length was set to two and the maximum repeat unit length was set to six. The minimum length of SSR was set to 15 and only sequences with perfect matches were selected.

### Data availability

The 454 EST data obtained in this study are available in the NCBI Sequence Read Archive under the accession SRA050067.

## Supporting Information

Figure S1 Multiple sequence alignment of switchgrass pyruvate transporters. Switchgrass pyruvate transporters localized to mitochondria and plastids versus pyruvate transporters localized to plastids only (A) or to motochondria only (B). MPpyrT refers to pyruvate transporters localized to mitochondria and plastids. PpyrT refers to pyruvate transporters localized to plastids. MpyrT refers to transporters localized to mitochondria. Multiple sequence alignments were conducted using the MAFFT version 6 (http://mafft.cbrc.jp/alignment/server/index.html). Flavaria BASS2 and BASS4 sequences were included in this analysis.
(TIF)

Figure S2 Multiple sequence alignment of switchgrass ESTs encoding NAD-malic enzyme. Thirty ESTs annotated as NAD malic enzyme was used for this analysis using the MAFFT version 6 (http://mafft.cbrc.jp/alignment/server/index.html). exCS refers to extracellular space. Thlk meb refers to thylakoid membrane. Plst refers to plastids. Mito refers to mitochondria.
(TIF)

Table S1 Dormancy related genes in switchgrass and Arabidopsis. Switchgrass ESTs from dormant seed libraries compared with Arabidopsis dormancy related genes [53].
(XLS)

Table S2 Primer sequences of switchgrass ESTs selected for RT-PCR analysis.
(DOC)
