# Supplementary figures and images for: Exploring the Switchgrass Transcriptome Using Second-Generation Sequencing Technology
Source: PLoS One. 2012 Mar 29;7(3):e34225. doi: 10.1371/journal.pone.0034225 (PMC3315583; doi:10.1371/journal.pone.0034225)

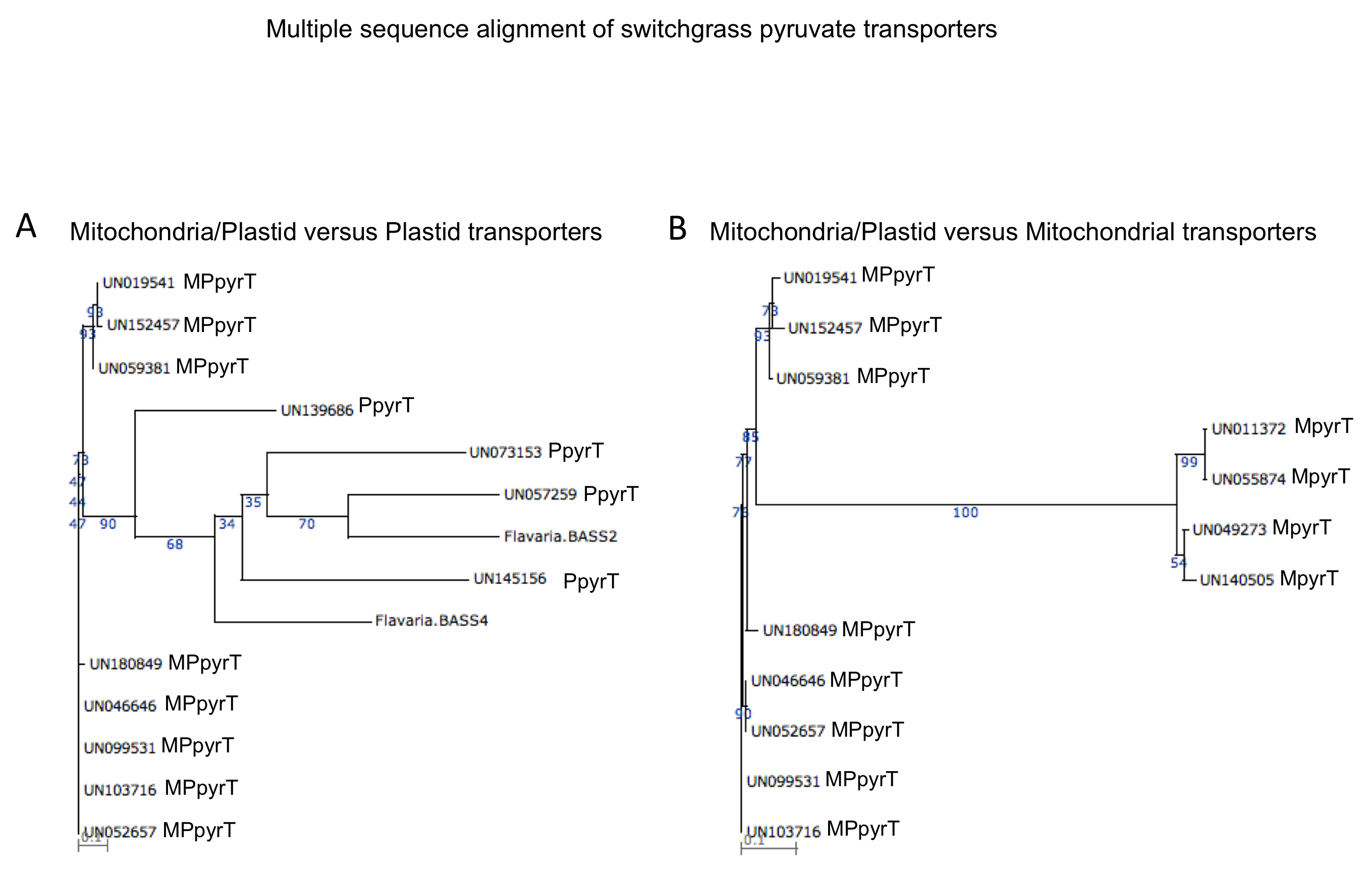

Supplement: Figure S1 — Multiple sequence alignment of switchgrass pyruvate transporters. Switchgrass pyruvate transporters localized to mitochondria and plastids versus pyruvate transporters localized to plastids only (A) or to motochondria only (B). MPpyrT refers to pyruvate transporters localized to mitochondria and plastids. PpyrT refers to pyruvate transporters localized to plastids. MpyrT refers to transporters localized to mitochondria. Multiple sequence alignments were conducted using the MAFFT version 6 (http://mafft.cbrc.jp/alignment/server/index.html). Flavaria BASS2 and BASS4 sequences were included in this analysis. (TIF) [file pone.0034225.s001.tif]

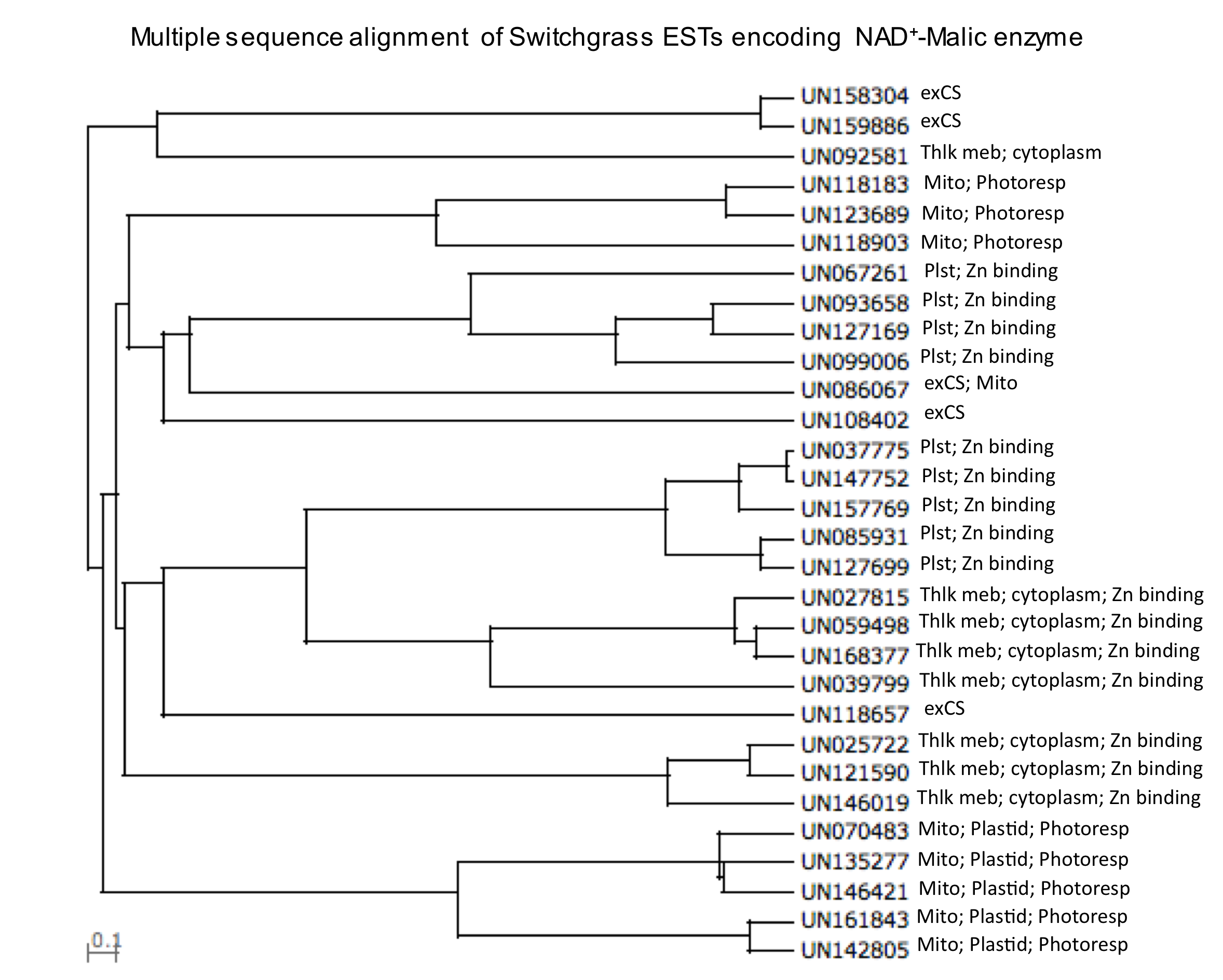

Supplement: Figure S2 — Multiple sequence alignment of switchgrass ESTs encoding NAD-malic enzyme. Thirty ESTs annotated as NAD malic enzyme was used for this analysis using the MAFFT version 6 (http://mafft.cbrc.jp/alignment/server/index.html). exCS refers to extracellular space. Thlk meb refers to thylakoid membrane. Plst refers to plastids. Mito refers to mitochondria. (TIF) [file pone.0034225.s002.tif]
