# Supplementary material for: Exploring the Switchgrass Transcriptome Using Second-Generation Sequencing Technology
Source: PLoS One. 2012 Mar 29;7(3):e34225. doi: 10.1371/journal.pone.0034225 (PMC3315583; doi:10.1371/journal.pone.0034225)
Supplement: Table S2 — Primer sequences of switchgrass ESTs selected for RT-PCR analysis. (DOC) [file pone.0034225.s004.doc]

Table S2. Primers for RT-PCR analysis of switchgrass genes.

| Primer Name | Primer Sequence (5’-3’) | Lane |
| --- | --- | --- |
| PvUni010595-F | AGCTTTCAAGGTCTTCGTCGCT | 1 |
| PvUni010595-R | AACAGCCCAGATCGTACGCTAA |  |
| PvUni046489-F | TAAGCCGCTGTTCCCATTTCCT | 2 |
| PvUni046489-R | AAACGCGGCGTGTTCGAATGAT |  |
| PvUni191496-F | AGAATCCAATGCCCGAAACCCA | 3 |
| PvUni191496-R | AAGCAGGGTCACCTTGTGTCCTTA |  |
| PvUni080121-F | TCCAAATCTGCCGAATCGCTCA | 4 |
| PvUni080121-R | TGTTTGAAGCACGCACAGAACC |  |
| PvUni320783-F | TGCAAGGTGGTCCTTGCTGATT | 5 |
| PvUni320783-R | TGCTGAATCCATGAGCAGGCAA |  |
| PvUni101034-F | AACCGAGTCTTAATTGGGCGCT | 6 |
| PvUni101034-R | ATTCGCAGTTTGCATCGGGTTG |  |
| PvUni235045-F | ATCCATGGGCAGGTTGAAGGTT | 7 |
| PvUni235045-R | TCGGAGTTTGCATCGGTTTGGT |  |
| PvUni104596-F | AGCGCTACCACAAGGCTAGAAT | 8 |
| PvUni104596-R | ACGCCAGTTGTCCATCACACTT |  |
| PvUni188933-F | TTCCAGGTCCACGCATGTTGAA | 9 |
| PvUni188933-R | ACCTGACATGGCATTTCCACGA |  |
| PvUni371915-F | ATACCTAGGCACCCAGAGACGA | 10 |
| PvUni371915-R | CTCATGGTATTCAGCAGGCTAGT |  |
| PvUni279096-F | TGCTTTCGCCGTTGGTGTTCTT | 11 |
| PvUni279096-R | TGATTGGGCGTAAAGCGTCTGT |  |
| PvUni383260-F | TGGGCGTTAAGTTGCAGGGTAT | 12 |
| PvUni383260-R | ATGAATTCACGAGGCGCTACCT |  |
| PvUni321268-F | ACACGAAGCAATCGGAAGTGGT | 13 |
| PvUni321268-R | TCGTGTTTGCAGAGTGCTGTGT |  |
| PvUni132986-F | TGCGAAGCAATCGGAAGTGGTT | 14 |
| PvUni132986-R | AACAGTCGCAGCCACCAGTTTA |  |
| PvUni296516-F | CCTAAGGAAGTGTGATGAACCC | 15 |
| PvUni296516-R | CGGGTTACTACTGATAACCAAACC |  |
| PvUni070139-F | ATCGTTGACACACCACACCACT | 16 |
| PvUni070139-R | ATTGGTTTCATCGGCCTGCTGT |  |
| PvUni035309-F | TTGAGTGAGCTTCGCACAAGCA | 17 |
| PvUni035309-R | TCGGTGCCATTGCATTCGGATA |  |
| PvUni094529-F | AAACCCAAGGTTTCCTCCGCAA | 18 |
| PvUni094529-R | AGGATCCTCGTGATTAGGCACT |  |
| PvUni357392-F | TGGCTCTAACGAGCTAATTTCACT | 19 |
| PvUni357392-R | TGAGTGAGTTTATGCTTCACTAATTG |  |
| PvUni250087-F | TGAGTAGCGATAATGCGGGTGA | 20 |
| PvUni250087-R | ACCACTTCCGATTGCTTCGTGT |  |
| PvUni199553-F | AAGGACGAGAGGTGGCCAGGTG | 21 |
| PvUni199553-R | TTATACTAGTGGTAGTCGGTTCCCGC |  |
| PvUni135373-F | TCCACGCAAGATGCACGAGAAGAT | 22 |
| PvUni135373-R | TTTCCCGCCAACGCACATATGGAA |  |
| PvUni049019-F | TGCTGCCTCTGACCAATGAAAC | 23 |
| PvUni049019-R | TGCGAGCTGTCTTTACTGTCCT |  |
| PvUni050011-F | GCCTTGGTTTGTTTGACTCCAG | 24 |
| PvUni050011-R | TGTGCAAGCAACCAGTCCATTC |  |
| PvUni050678-F | CGACAACAGGTTCAATGCCGTA | 25 |
| PvUni050678-R | GCTTCTGCTTGAGTTGCATGTG |  |
| PvUni051344-F | GCTTTGGTGCGTCATTTCGATG | 26 |
| PvUni051344-R | ATGAGAGGGATTGGGCGTCTTA |  |
| PvUni049849-F | TATCCTGTTCCAGCATTCCCGT | 27 |
| PvUni049849-R | GGAGGAAACTACACATCCAGCA |  |
| PvUni049879-F | AGCTAGTTCCTGAGATGTGCGT | 28 |
| PvUni049879-R | TGCATGATCGCTGTCATCATCT |  |
| PvUni059368-F | GCATTGCCGCACTTACCTACAA | 29 |
| PvUni059368-R | CTAACCAATCTGCGCAAGCGTT |  |
| PvUni062816-F | CGCCTCCAGTACGTTCATCATT | 30 |
| PvUni062816-R | TGCTAAAGCTGTTTCCAAGCCG |  |
| PvUni071721-F | CCCATTAGCGCAAGATGCTACT | 31 |
| PvUni071721-R | AGCTGCTTCATATCGCCGTTGA |  |
| PvUni093423-F | AGGAAGAAGAAGGAGGAGAAGG | 32 |
| PvUni093423-R | GCCAAATGGACCCAGGAAGA |  |
| PvUni096348-F | ATTGGAGCACAGGTTCAAGCTC | 33 |
| PvUni096348-R | ATAGCTTCACCACCTCCCAGTT |  |
| PvUni101024-F | GGTTGTTGCTGAGCTTGAACCA | 34 |
| PvUni101024-R | AACTCCAGTTACGAGCGCTTTC |  |
| PvUni233397-F | GCGAGCGTTCCGATTTGGTAAA | 35 |
| PvUni233397-R | AACGAAGATCTCGGCATCACCA |  |
| PvUni281658-F | TCATGAGCATCTTGTGGCAACC | 36 |
| PvUni281658-R | GACGACGATGCGAAGCAATGAT |  |
| PvUni381911-F | AGGAACCTTGACCTGTCTCGTT | 37 |
| PvUni381911-R | AAGCGAACGGATGTTGTTGCAG |  |
| PvUni088831-F | TGGGTTGTCTTGGGTTTCCTCT | 38 |
| PvUni088831-R | TTCTCTTGCGCGAGACTAACCT |  |
| PvUni244961-F | TGCATGCGTGCATGAGAACGAA | 39 |
| PvUni244961-R | ACCCATGTGTGTGTGTGTGTGT |  |
| PvUni346605-F | GGCACTATGAAACGCGACCAAA | 40 |
| PvUni346605-R | GTGCCAGAAAGTTGCATCGGAA |  |
| PvUni097040-F | AGCCGGACAAGAGAAAGAAGGA | 41 |
| PvUni097040-R | TTTGTGAGGGCGATGGTGACTA |  |
| PvUni125942-F | GGTGGACATGTGCAAAGGCATA | 42 |
| PvUni125942-R | ATTCAGGGAGCCACTTTGGAGA |  |
| Tb1-F | CCAACCTACACCAACTTGAACA | 43 |
| Tb1-R | TGGACAGTTTCTCTTGGTCTTGA |  |

The F and R following the PvUnigene identifier refer to forward and reverse.

Lane column refers to the numbers in the figure 8 showing the RT-PCR amplification of genes using the corresponding primer pairs.

Tb1 refers to the switchgrass homolog of the maize teosinte branched 1 gene.
